# Factors associated with malaria parasitaemia among children under 5 years in Uganda: a secondary data analysis of the 2014 Malaria Indicator Survey dataset

**DOI:** 10.1186/s12936-017-1847-3

**Published:** 2017-05-08

**Authors:** Humphrey Wanzira, Henry Katamba, Allen Eva Okullo, Bosco Agaba, Mathias Kasule, Denis Rubahika

**Affiliations:** 1 National Malaria Control Programme, Ministry of Health, Kampala, Uganda; 2 Makerere University, School of Public Health, Fellowship Programme, Kampala, Uganda

**Keywords:** Malaria parasitaemia, Anaemia, Children under 5 years

## Abstract

**Background:**

In the midst of success with malaria reduction in Uganda, there are areas that still have high prevalence of malaria parasitaemia. This project aimed at investigating factors associated with this prevalence and its relationship with anaemia.

**Methods:**

This is a secondary data analysis of the 2014 Malaria Indicator Survey dataset of children under 5 years. All had a blood sample taken by finger or heel prick for determination of malaria parasitaemia and estimation of haemoglobin level for anaemia status. The main outcome was the presence of malaria parasitaemia by microscopy and independent variables included: age, gender, residence (urban vs rural), use of a long-lasting, insecticidal-treated net, indoor residual spraying (IRS) of household in the past 6 months, mother’s highest education level, mother heard malaria prevention message in the past 6 months, and household wealth status.

**Results:**

The analysis included 4930 children and of these, 938 (19.04%: 95% CI 16.63–21.71) tested positive for malaria parasites. Malaria parasite prevalence significantly increased from 11.08 (95% CI 9.12–13.40) among children with no anaemia to 50.99% (95% CI 39.13–62.74) with severe anaemia (Chi-square p-value = 0.001). Additionally, prevalence significantly rose from the youngest age group (under 6 months) by 1.62 times (95% CI 1.04–2.52, p = 0.033) among the age group of 7–12 months and to four times (95% CI 2.57–6.45, p = 0.001) among those who were between 49 and 59 months. The following were associated with reduced parasitaemia: IRS use (AOR 0.23 [0.08–0.61], p = 0.004), educated mothers (primary AOR 0.75 [0.59–0.96], p = 0.023 to tertiary AOR 0.11 [0.02–0.53], 0.006), mother heard malaria message (AOR 0.78 [0.62–0.99], p = 0.037), and wealthier households (richest AOR 0.17 [0.08–0.36], p = 0.001).

**Conclusions:**

Increasing malaria parasite prevalence among children under 5 years is still related to increasing age and severity of anaemia even in the context of decreasing malaria prevalence. Designing interventions that include the use of IRS and behaviour change communication tailored to include older children, especially in areas with high malaria prevalence, could be of added value. All this should be done in an environment that improves the socio-economic status and equity of such populations.

## Background

Uganda is one of the sub-Saharan African countries where malaria is still endemic in over 90% of the countries regions [1–3]. In such settings, malaria is a major contributor to anaemia, with severe anaemia (defined as a haemoglobin level below 8 g/dl), as the main manifestation of complicated malaria [4, 5]. Both conditions are known to contribute to the huge burden of morbidity and mortality, especially among children under 5 years of age [6, 7]. According to the National Malaria Control Programme (NMCP), malaria alone has been shown to contribute to between 30 and 50% of outpatient visits, 15–20% of hospital admissions and 20% of hospital deaths with most of this burden borne by children under 5 years and pregnant women [1]. Anaemia alone also affects over 50% of individuals in the same populations [7], making both illnesses conditions of great public health concern.

So far, the NMCP has led a number of efforts to reduce this malaria burden, which include universal coverage of long-lasting insecticidal nets (LLINs), indoor residual spraying (IRS), case management, and intermittent preventive therapy in pregnant women [1]. Already there is great success on this front according to the recently released 2014 Malaria Indicator Survey (MIS) report [8], which showed that parasite prevalence by microscopy among children under 5 years had reduced from 42% in the 2009 MIS report to the present 19%, a 23 point decrease in a period of approximately 5 years. The same success among this age group followed the prevalence of anaemia, with 10% of children estimated to have had severe anaemia (haemoglobin (Hb) <8 g/dl) in 2009 MIS compared to 5% in the 2014 MIS, a 50% reduction in prevalence. However, the same report clearly indicates that the parasite prevalence reduction is not homogenous, with some regions having prevalence as high as 36% in the East Central region, while the urban capital city of Kampala had estimates as low as 0.4%. This is an important issue in the fight against malaria because areas of high malaria parasite prevalence are potentially the epicentre of a surge of malaria infections towards areas with lower malaria burden, whenever conditions are favourable for mosquito breeding [9]. It is therefore important to understand the reasons that contribute to high prevalence of malaria parasitaemia in some areas and not in the others. This is the starting point in the planning and focused implementation of evidence-proven efforts tailored towards such regions. A number of studies have been conducted exploring factors associated with malaria parasite prevalence, ranging from individual factors such as age and haemoglobinopathies [10, 11], use of prevention strategies such as LLINs and IRS [12, 13] and the effects of environment and social-economic status [14, 15]. However, there is limited evidence exploring this relationship with a large and representative sample population, especially in the Ugandan context. Using the 2014 MIS dataset, this project sought to examine factors associated with malaria parasitaemia among children under 5 years of age and to explore the relationship between malaria parasite prevalence and anaemia in the context of decreasing trends of malaria burden.

## Methods

This was a secondary data analysis project using the 2014 MIS datasets, a description of which is given below.

### The 2014 Malaria Indicator Survey (MIS)

The survey was conducted during December 2014 and January 2015 [8]. Households were selected using a stratified, two-stage, cluster design from 210 enumeration areas, representing all the regions of the country, with 44 urban areas and 166 rural areas. An EA was defined as a natural village in rural areas and a city block in urban areas. In the first stage, 20 sampling strata (derived from ten regional domains: Central 1 and 2, East Central, Kampala, Mid-Northern, Mid-Western, Mid-Eastern, South-Western and West Nile) were created and EAs were selected independently from each stratum by a probability-proportional-to-size selection. In the selected EAs, a complete listing of households and a mapping exercise was conducted in November 2014, with the resulting list of households serving as the sampling frame for the selection of households in the second stage. The average EA size was 94 households in urban areas and 77 households in rural areas, with an overall average size of 80 households per EA. In the second stage of the selection process, 28 households were selected in each EA by equal probability systematic sampling. A total of 5802 households were selected for the 2014 MIS, of which 5494 were occupied. Of the occupied households, 5345 were successfully interviewed, yielding a response rate of 97%. The response rate among households in rural areas was slightly higher (98%) than the response rate in urban areas (96%). The main reason for non-response was failure to find individuals at home despite up to four repeated visits to the household. Two questionnaires were used to collect survey data, the household questionnaire (administered to all household heads) and women’s questionnaire (administered to all women aged 15–45 years in a selected household). Informed consent was obtained from all participating heads of households and women of childbearing age in the households that participated in the survey.

For the specifics of this study, information from two 2014 MIS datasets was used: children under 5 years and individual member datasets. The children under 5 years dataset contained information related to a child’s individual characteristics, health and treatment of childhood diseases. The individual member dataset had one record for every household member and included additional variables such as mother’s highest education attainment, mother’s treatment-seeking behaviour, use of LLINs, use of IRS and household wealth.

### Laboratory tests for anaemia and malaria

During the survey, capillary blood samples were taken from the fingers or heels of children age 0 up to 59 months. Testing for anaemia was done by Hb analysis which was carried out on-site using a battery-operated portable HemoCue analyser, which produces a result in less than 1 min. Malaria testing by microscopy involved preparing both the thick and thin blood smears using 2% Giemsa stain. As a quality control measure, each smear was read by two independent expert microscopists who were not involved with the participant respondent interview. A third reader was only involved to settle parasite presence discrepancy between the two readers. A positive smear parasite density (parasites/µl) was estimated by counting the number of asexual parasites per 200 white blood cells, assuming a white blood cell count of 8000 cells/µl. A smear was only considered negative if no parasites were seen after viewing 100 high-powered fields. Thin smears of positive thick smears were read to determine the species of *Plasmodium* parasite. Additionally, a malaria rapid diagnostic test was performed in order to offer prompt treatment to participants that were positive for malaria.

### Study variables

The dependent variables of interest in this analysis were the presence of malaria parasites by microscopy and prevalence of anaemia among children under 5 years of age. Using similar cut-offs as the 2014 MIS [8], anaemia was grouped into four categories that included not anaemic (Hb ≥11 g/dl), mild anaemia (Hb = 10.9–10 g/dl), moderate anaemia (Hb = 9.9–8 g/dl), and severe anaemia (Hb <8 g/dl).

Independent variables considered for presence of malaria parasites included: age, gender, residence (urban vs rural), use of an LLIN, IRS of household in the past 6 months, mother’s highest education level attained, if mother has heard malaria prevention message in the past 6 months, and the extent of the household wealth.

### Data analysis

Stata version 14 (Statcorp, College Station, TX, USA) was used for all data analysis. In order to create the dataset for this analysis, two 2014 MIS datasets: the children and individual member datasets were merged. Due to the non-proportional allocation of sample in the different regional domains at the second sampling stage (28 households were selected in each EA by equal probability systematic sampling), the sample was not self-weighting. Weighting factors were calculated based on the population of the selected regional domains and added to the MIS datasets so that any results with the regional weight factored into it would be representative at national and regional level as well as survey domain level. Details of how the weighting for the different regional domains was estimated are available in the 2014 MIS report [8]. Therefore, for this study, only weighted survey data are presented in this manuscript. The distributions of study participant baseline characteristics were presented as frequencies with respective proportions. A Chi-square test was used to estimate the level of significance for the differences in proportion of malaria parasite prevalence among the four categories of anaemia. A multivariate logistic regression model with a survey function was used to assess for the factors associated with the presence of malaria parasitaemia to derive first the crude and then the adjusted odds ratio with its respective confidence interval. Variables included in the adjusted model were: age, gender, residence, LLIN use, IRS use, mother’s education level, if mother heard malaria message, and household wealth. In all analyses, a p-value of <0.05 was taken as statistically significant.

### Ethical considerations

The 2014 MIS was approved by two ethical review bodies that included the Makerere University School of Biomedical Sciences Higher Degrees Research and Ethics Committee (SBS-HDREC) and the Uganda National Council for Science and Technology (UNCST). All participants interviewed gave their written informed consent to participate in the 2014 MIS in addition to granting permission that information from survey could be published. Furthermore, written informed consent was sought from the mother or guardian of a child before any blood sample was taken. The data used in this analysis were anonymous with no individual names of participants captured.

## Results

### Participants’ characteristics

**Fig. 1:**
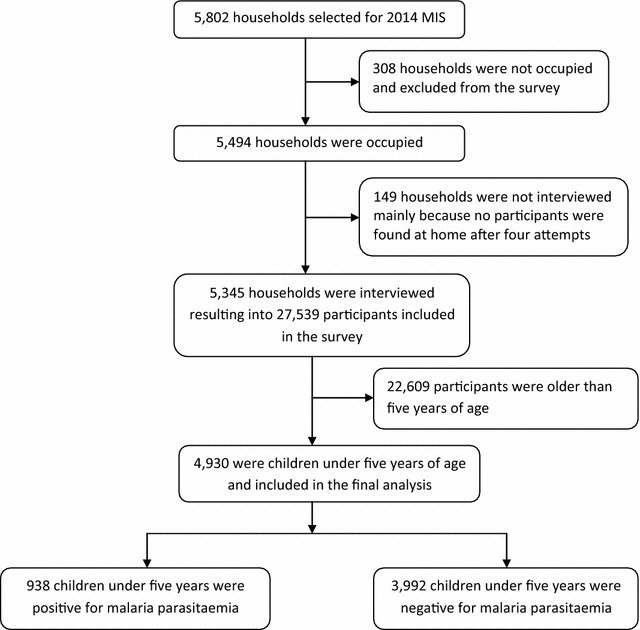
Study profile

A total of 4930 children under 5 years old were included in this analysis (Fig. 1) of which 4156 (84.29%) of respondents were mothers of the children. Slightly more children were females than males (2540 [51.52%] vs 2390 [48.48%]) while their age (months) ranged from 0 to 59 months with a mean age (SD) of 30.18 (16.94). Table 1 shows that children between age group 0–6 months were the fewest, 491 (9.95%), while those in the age group 37–48 months were the most, at 1081 (21.94%). Most of the children [4150/4930 (84.18%)] were from a rural setting and in the ten sampled regions. Just over half of the children’s mothers had attained primary education at 2514 (60.79%), while 134 (3.24%) attained tertiary education. The majority of children were from the poorest households at 1108 (22.48%), while the least were from the richest households at 836 (16.96%).

**Table 1:** Baseline characteristics of study participants

Characteristic	Distribution of participants	
	Total population, N = 4930	Percentage
Age categories (months)		
0–6	491	9.95
7–12	492	9.97
13–24	984	19.97
25–36	985	19.97
37–48	1081	21.94
49–59	897	18.19
Gender		
Male	2390	48.48
Female	2540	51.52
Residence		
Urban	780	15.82
Rural	4150	84.18
Region		
Central 1	536	10.88
Central 2	529	10.72
East central	564	11.43
Kampala	183	3.70
Mid-North	556	11.29
Mid-Western	629	12.77
Mid-Eastern	534	10.83
North-East	499	10.12
South-Western	522	10.59
West-Nile	378	7.67
Mother’s education^a^		
No education	755	18.26
Primary	2514	60.79
Secondary	732	17.71
Tertiary	134	3.24
Household wealth		
Poorest	1108	22.48
Poorer	1078	21.87
Middle	1000	20.27
Richer	908	18.42
Richest	836	16.96

^a^ 795 Missing mother’s education status

### Prevalence of malaria parasitaemia

Overall, 938 (19.04%: 95% CI [16.63–21.71]) children under 5 years, out of the 4930 in the sample had malaria parasitaemia by microscopy, on the day of the survey; 95.65% of all these cases of parasitaemia were due to *Plasmodium falciparum*. Presence of fever in the 2 weeks prior to the survey was reported in 285 out of the 938 (30.33%) children diagnosed with malaria parasitaemia.

### Relationship between malaria parasite prevalence and anaemia

**Fig. 2:**
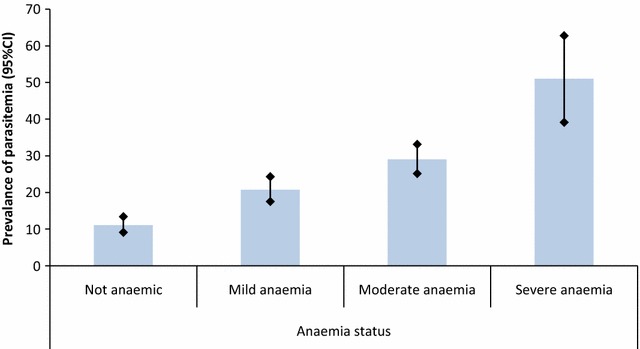
Percentage of malaria parasite prevalence among the different categories of anaemia

Figure 2 shows that 2296 (46.65%) children were not anaemic, 1188 (24.13%) had mild anaemia, 1349 (27.40%) had moderate anaemia, and 89 (1.82%) had severe anaemia. Malaria parasite prevalence significantly increased from 11.08 (95% CI 9.12–13.40) among those with no anaemia to 20.73% (95% CI 17.54–24.31) in children with mild anaemia to 28.98 (95% CI 25.15–33.13) with moderate anaemia, and peaked at 50.99% (95% CI 39.13–62.74) in the category with severe anaemia (Chi-square p-value = 0.001).

### Factors associated with malaria parasitaemia

Using the age group up to 6 months as a reference category, there was a significant rising trend in odds of having malaria parasitaemia with each increasing age category, from 1.62 times among those aged 7–12 months to four times among those who were between 49 and 59 months (Table 2). The odds of having parasitaemia among children from households that had received IRS were significantly reduced by 77% as compared to those that had not received IRS, however, the opposite was observed from LLIN use, with the odds among children who had used an LLIN significantly increasing by 1.33 times as compared to those who did not use one. The higher the mother’s education attained directly correlated with a significant reduction in the odds of her children having malaria parasitaemia, from a 25% reduction among those with primary education to 89% reduction among those with tertiary education, using those with no education as the reference category. Similarly, children from wealthier households had reduced odds of having parasitaemia, with a significant reduction trend with increasing wealth from 30% in the poorer households to 89% in the richest households, using the poorest households as a reference. There were no statistically significant differences in odds of having parasitaemia for gender, residence (urban and rural) and region.

**Table 2:** Factors associated with malaria parasitaemia

Variables	Malaria parasitaemia by microscopy		Odds ratio (95% CI)		p-value
	Negative, N = 3992	Positive, N = 938	Crude OR	Adjusted OR^a^	
Age categories (months)					
0–6	451 (91.97)	39 (8.03)	1	1	
7–12	437 (88.92)	54 (11.08)	1.43 (0.95–2.14)	1.62 (1.04–2.52)	0.033^*^
13–24	827 (84.03)	157 (15.97)	2.18 (1.51–3.13)	2.20 (1.47–3.29)	0.001^*^
25–36	767 (77.89)	218 (22.11)	3.25 (2.22–4.76)	3.47 (2.32–5.20)	0.001^*^
37–48	844 (78.03)	238 (21.97)	3.23 (2.27–4.58)	3.69 (2.47–5.50)	0.001^*^
49–59	665 (74.11)	232 (25.89)	4.00 (2.72–5.88)	4.01 (2.57–6.45)	0.001^*^
Gender					
Male	1936 (80.99)	454 (19.01)			
Female	2056 (80.94)	484 (19.06)	1.00 (0.82–1.22)	1.04 (0.82–1.32)	0.764
Residence					
Urban	730 (93.65)	50 (6.35)			
Rural	3261 (78.58)	889 (21.42)	4.02 (1.87–8.65)	1.74 (0.92–3.29)	0.089
Use of LLIN					
No	1056 (83.72)	205 (16.28)			
Yes	2936 (80.01)	733 (19.99)	1.29 (1.02–1.62)	1.33 (1.04–1.71)	0.026^*^
IRS use					
No	3742 (80.35)	915 (19.65)			
Yes	2447 (92.77)	19 (7.23)	0.32 (0.12–0.83)	0.23 (0.08–0.61)	0.004^*^
Mother’s education					
No education	549 (72.70)	206 (27.30)	1	1	
Primary	2036 (80.98)	478 (19.02)	0.63 (0.50–0.79)	0.75 (0.59–0.96)	0.023^*^
Secondary	660 (90.12)	72 (9.88)	0.29 (0.21–0.40)	0.61 (0.43–0.86)	0.005^*^
Tertiary	133 (98.92)	1 (1.08)	0.03 (0.01–0.13)	0.11 (0.02–0.53)	0.006^*^
Mother heard malaria message					
No	1266 (77.63)	365 (22.37)			
Yes	2315 (83.28)	465 (16.72)	0.70 (0.56–0.86)	0.78 (0.62–0.99)	0.037^*^
Household wealth					
Poorest	799 (72.11)	309 (27.89)	1	1	
Poorer	831 (77.07)	247 (22.89)	0.77 (0.58–1.02)	0.70 (0.50–0.99)	0.043^*^
Middle	774 (77.44)	225 (22.56)	0.75 (0.53–1.07)	0.75 (0.50–1.12)	0.157
Richer	784 (86.26)	124 (13.56)	0.41 (0.29–0.59)	0.40 (0.27–0.61)	0.001^*^
Richest	804 (96.16)	32 (3.84)	0.10 (0.06–0.18)	0.17 (0.08–0.36)	0.001^*^

* p ≤ 0.05

^a^ Factors adjusted for included age, gender, residence, use of LLIN, use of IRS, mother’s education, if mother heard a malaria message, and household wealth

## Discussion

This analysis has identified a number of factors associated with malaria parasitaemia that could be considered during the design and implementation of interventions to areas with high malaria prevalence. The finding that malaria parasitaemia prevalence increases with increasing age of a child, coupled with a reducing occurrence of the typical malaria fever symptom is in agreement with results of other studies that have shown this similar trend [16–18]. Additionally, there is already a scientific explanation for this occurrence: that children in areas of high malaria transmission intensity develop age-related immunity due to the continuous exposure to infective mosquito bites [19, 20]. This immunity develops first against complicated, then uncomplicated malaria and later on against malaria parasitaemia. This explains the state that older children will more likely have malaria parasites without developing clinical disease, unlike their younger counterparts who have immature immunity and are still battling severe clinical disease. Knowledge of this information is useful during the design of interventions specifically for use in such areas, which could include a package focused on older children even when they are not clinical ill, since such children already act as malaria parasite reservoirs and are part of the drivers of malaria infections [21, 22]. However, this analysis did not seek to identify hot spots of parasitaemia since the dataset did not include global position system (GPS) data in addition to missing parasite density information.

There is great success with the decline of the prevalence of malaria parasitaemia and anaemia, however, this analysis has shown that even in this situation, there is still a strong association between the two conditions. There is already strong evidence that malaria is one of the major causes of anaemia in malaria-endemic areas with severe anaemia as a most common malaria complication in these settings [23–26], the pathogenesis of which is not clearly understood but is thought to be a combination of haemolysis of red blood cells, activation of the reticulo-endothelial system leading to destruction of uninfected red cells, auto-immune destruction of red cells, defective production of erythropoietin and bone marrow depression [27, 28]. Therefore, in areas of reducing malaria burden, malaria would no longer be the major cause of anaemia and therefore this association is expected to weaken, which is still not the case in this setting.

The proportion of malaria parasitaemia among children with anaemia increases with increasing severity of anaemia, an indirect indication that parasitaemia is still a major contributor of anaemia especially among those with severe anaemia, as shown by other studies [12, 29]. This finding emphasizes the continued strategy of aggressively controlling malaria and treatment of anaemia to reduce morbidity and mortality from these two conditions even at the time when parasitaemia has drastically reduced.

Of importance in the fight against malaria, there are two current malaria control strategies that are associated with reducing malaria parasite prevalence, the use of IRS and behaviour change communication (BCC). Just as in previous studies [12, 29], the spraying of households with an insecticide showed a significant 77% reduction of malaria prevalence among children from households that had received IRS in the 6 months prior to the survey compared to those who had not. However, the use of an LLIN peculiarly did the opposite, with children who used an LLIN more likely to have parasitaemia, a finding that contradicts what has been reported elsewhere [13]. This raises important questions regarding proper LLIN use and reporting during the survey, in addition to their efficacy, which unfortunately was not adequately explored in the MIS. However, there is the possibility that at the time of the survey, the children in areas with high malaria burden, already with a high malaria prevalence, had a greater incentive to use an LLIN or that high malaria prevalence preceded LLIN distribution and use. One of the weaknesses of this cross-sectional study design is its inability to estimate the time sequence of events, which could have ruled out this assumption.

The second strategy is the use of BCC, which is supported by the finding that children from mothers who had heard malaria-related messages in the 6 months prior to the survey had 22% reduced odds of having malaria parasites. The use of BCC in the prevention of malaria is already well documented [30–32], and therefore the addition of a package that emphasizes and creates awareness directed towards older children who are seemingly well, for example embedded in a school programme, could be of added advantage.

The significance of a higher education attainment of a child’s mother and a better wealth status of a household in which a child dwells in reducing the risk of a child’s ill health, including malaria, is well published [33–35]. Mothers who have attained a higher education have greater exposure to means and methods of living a healthier life, and specifically to prevention and treatment of malaria, which they pass on to their children, in the same way as individuals in wealthier households [11]. This realization emphasizes the fact that malaria control and ultimately eradication is not an isolated effort of a malaria control programme, but part of a holistic approach of improving education and socio-economic status of a population [36].

Due to the fact this was an observational study design, there is the possibility that some factors associated with malaria parasitaemia were not considered in this analysis because of reliance on variables collected only during the survey.

## Conclusions

This project has shown that malaria parasitaemia among children under 5 years in this setting is still related to increasing age and anaemia severity even in the context of decreasing trends of parasitaemia, suggesting that designing interventions that include older children who are seemingly well could be of added value. The use of IRS and BCC should be part of these interventions, especially in areas of high malaria prevalence. All this should be a holistic approach, which includes improving the socio-economic status and equity of such populations. However, the use of LLINs was not associated with a reduction in prevalence suggesting that further studies to explore proper LLIN use, reporting and efficacy in such settings are recommended. These findings are applicable to the Ugandan population since the 2014 MIS had an unbiased sampled population covering all regions of the country.

## Abbreviations

BCC: behaviour change communication
EA: enumeration area
LLIN: long-lasting insecticide-treated bed nets
MIS: Malaria Indicator Survey
NMCP: National Malaria Control Programme
SBS-HDREC: Makerere University School of Biomedical Sciences Higher Degrees Research and Ethics Committee
UNCST: Uganda National Council for Science and Technology
WHO: World Health Organization
